# Simultaneous knockdown of six non-family genes using a single synthetic RNAi fragment in *Arabidopsis thaliana*

**DOI:** 10.1186/s13007-016-0116-8

**Published:** 2016-02-17

**Authors:** Olaf Czarnecki, Anthony C. Bryan, Sara S. Jawdy, Xiaohan Yang, Zong-Ming Cheng, Jin-Gui Chen, Gerald A. Tuskan

**Affiliations:** Biosciences Division, Oak Ridge National Laboratory, Oak Ridge, TN 37831 USA; Department of Plant Sciences, University of Tennessee, Knoxville, TN 37996 USA; KWS SAAT SE, Grimsehlstraße 31, 37555 Einbeck, Germany

## Abstract

**Background:**

Genetic engineering of plants that results in successful establishment of new biochemical or regulatory pathways requires stable introduction of one or more genes into the plant genome. It might also be necessary to down-regulate or turn off expression of endogenous genes in order to reduce activity of competing pathways. An established way to knockdown gene expression in plants is expressing a hairpin-RNAi construct, eventually leading to degradation of a specifically targeted mRNA. Knockdown of multiple genes that do not share homologous sequences is still challenging and involves either sophisticated cloning strategies to create vectors with different serial expression constructs or multiple transformation events that is often restricted by a lack of available transformation markers.

**Results:**

Synthetic RNAi fragments were assembled in yeast carrying homologous sequences to six or seven non-family genes and introduced into pAGRIKOLA. Transformation of *Arabidopsis thaliana* and subsequent expression analysis of targeted genes proved efficient knockdown of all target genes.

**Conclusions:**

We present a simple and cost-effective method to create constructs to simultaneously knockdown multiple non-family genes or genes that do not share sequence homology. The presented method can be applied in plant and animal synthetic biology as well as traditional plant and animal genetic engineering.

**Electronic supplementary material:**

The online version of this article (doi:10.1186/s13007-016-0116-8) contains supplementary material, which is available to authorized users.

## Background

Targeted gene knockdown by RNA interference (RNAi) has become a powerful tool for genetic research and biotechnology in eukaryotes. Originally discovered in the nematode *Caenorhabditis elegans* [1], details of the molecular mechanisms underlying the gene silencing caused by double-stranded RNA (dsRNA) have been elucidated within the last two decades [reviewed in 2]. Briefly, dsRNA molecules are cut in pieces of 21–23 nucleotides termed small interfering RNA (siRNA), by RNAse III family endoribonucleases named DICER. ARGONAUTE proteins acting in complex with DICER as RNA-induced silencing complex (RISC) mediate unwinding of siRNA molecules where the passenger strand is released and degraded and the guide strand serves as recognition pattern to bind complementary single-stranded RNA (ssRNA) molecules (mRNA). The endonuclease activity of ARGONAUTE results in specific degradation of the homologous ssRNA molecule and RISC eventually binds to other target molecules [2–6].

The origin of dsRNA molecules can be endogenous or exogenous. Endogenous dsRNA molecules have been identified in both plants and animals and are derived from transposable elements, transcripts containing short inverted repeats or natural antisense transcripts [7–11]. Exogenous dsRNA molecules, on the other hand, are derived from viruses and trigger host defense mechanisms against viral RNA [12, 13].

Soon after its discovery, RNAi became an important tool for reverse genetics in plants, as it enables targeted gene knockdown. There are at least three advantages of RNAi: (1) the ability to knock down gene family members that share homologous sequences or orthologous genes in polyploid organisms relative to other methods (e.g., T-DNA insertion or EMS mutagenesis), (2) the genetically dominant mode of action of RNAi and (3) relative easiness to create transgenic plants expressing RNAi transgenes, depending on the availability of suitable RNAi plasmids and on the transformability of the organisms of interest [14–17].

In plant biotechnology, RNAi is induced by constitutive, induced or spatial/tissue-specific expression of a target gene fragment cloned as a tandem inverted repeat separated by a hairpin forming intron sequence. The transcript creates a hairpin RNA (hpRNA) that then serves as template for the RNAi machinery [18]. There are several routine plant RNAi transformation vectors available, e.g., pHANNIBAL [19], pHELLSGATE [19], pAGRIKOLA [20], pOpOff [21] and the pFGC and pGSA series [22].

There has been a tremendous amount of literature describing the modulation of transcript abundance of single genes in plants using RNAi and/or over-expression constructs. Engineering of complex metabolic pathways, however, often requires controlling more than one gene in the same or interconnected pathways [23, 24]. Current strategies to create multiple transgene plants involve sexual crossing or co-transformation and retransformation. Golden rice is an example where the entire β-carotene biosynthetic pathway was introduced into the rice endosperm by a single transformation event using three different vectors [25]. There have been several attempts to improve and create stable plant transformation vectors and cloning strategies for gene stacking, such as sophisticated use of recombinases [26–29]. Still, successful development of new or synthetic biochemical pathways in plants might not only require expression of multiple genes but also knockdown of more than one endogenous gene. In animals and cell lines, serial expression of several small hairpin RNAs (shRNA) in virus derived vectors is an established method to achieve multiple gene knockdown, but the cloning strategies to create the vectors are rather complex and time consuming [30–34]. Recently, genome editing has become an important tool to achieve a knockout of one or multiple genes in eukaryotes. For instance, TALEN or CRISPR/Cas based approaches allow targeted and highly efficient introduction of premature stop codons in the open reading frame of target genes [35–38]. However, reduction of target gene expression or gene product activity by genome editing is challenging without detailed knowledge of regulatory elements affecting gene expression or amino acid substitutions affecting protein activity.

Here we present a new consolidated method to knockdown alternant non-family genes using a single artificial gene fragment cloned in a binary plant RNAi vector. As proof-of-concept, six- and seven-gene-RNAi vectors were introduced into *Arabidopsis thaliana* with successful down regulation of the target genes. Our technology has applications in plant and animal synthetic biology as well as traditional plant and animal genetic engineering.

## Results and discussion

### Cloning of multiple target RNAi vectors

**Fig. 1:**
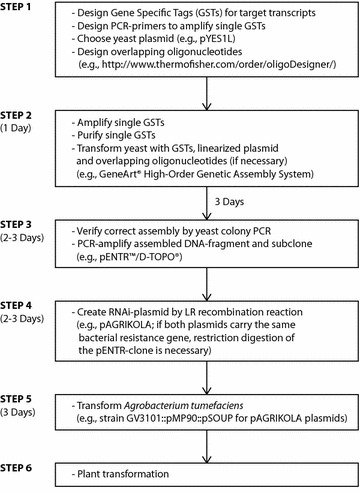
Flowchart illustrating steps and approximate time needed to create synthetic hpRNAi constructs by TAR in order to generate transgenic *Arabidopsis* plants expressing a single synthetic hpRNAi construct to knockdown different genes simultaneously. Cloning of the synthetic RNAi construct using commercially available kits is relatively cost-effective and constructs are ready for plant transformation within 2 weeks

Based on the principle that hpRNA-induced RNAi uses 21–23 bp siRNA fragments to degrade target transcripts, we developed a hypothesis that expression of a single hpRNA fragment consisting of different gene specific tags (GSTs) in Arabidopsis will eventually result in a set of siRNAs specifically degrading all transcripts targeted by the respective GSTs. To prove this hypothesis, synthetic DNA fragments consisting of six or seven alternant GSTs were assembled by means of transformation-associated recombination [TAR, 39] in *Saccharomyces cerevisiae*. This method is based on homologous recombination after simultaneous uptake of a linear double-stranded vector and double-stranded PCR products that share sequence homology or overlapping single-stranded oligonucleotides in a single transformation event [40, 41]. It has widely been used in biotechnology, including assembly of synthetic bacterial genomes [42, 43], cloning of the human mitochondrial genome [44] and joining of unrelated DNA fragments during plasmid assembly [45]. We applied this method to assemble unrelated GSTs of different target genes to synthetic hpRNA fragments (Fig. 1).

We selected seven target genes for this proof-of-concept study to be downregulated simultaneously in *A. thaliana*. Candidate genes do not share sequence homologies and are therefore considered as non-family genes (Table 1). Alignments of cDNA do not reveal any 21–23 nucleotides stretches of identical sequence (Additional 1: Fig. S1) and we assume that siRNA of one target gene do not interfere with other target genes. To our best knowledge candidate genes do not act in the same biological pathways to avoid transcript levels being affected by feedback mechanisms or pleiotropic effects. Moreover, phenotypes of knockout or loss-of-function mutants of the selected genes have been described previously [reviewed in 46]. *AtHY2* (ELONGATED HYPOCOTYL 2) is involved in biosynthesis of heme and phytochromobillin, the phytochrome chromophor and mutants develop elongated hypocotyls. *AtTRY* (TRIPTYCHON) is a negative regulator of trichome development and a knockout causes visible trichome clusters on leaves. *AtLNG1* (LONGIFOLIA1) regulates longitudinal cell elongation resulting in characteristic long leaf shapes when knocked out. *AtNPQ1* (NON-PHOTOCHEMICAL QUENCHING 1) is a violaxanthin deepoxidase involved in xanthophyll cycle and the lack of zeaxanthin caused by a gene knockout leads to a stress phenotype under high light intensities. *AtSEX1* (STARCH EXCESS 1) is required for starch degradation in leaves and knockout mutants accumulate large amounts of starch in adult leaves. *AtMAX3* (MORE AXILLARY BRANCHING 3) plays a role in strigolactone biosynthesis and *max3* mutants display a bushy appearance and increased shoot branching. Finally, *AtGUN4* (GENOME UNCOUPLED 4) is a regulator of chlorophyll biosynthesis in chloroplasts and the *gun4*-*1* mutant showing reduced GUN4 activity suffers from reduced chlorophyll contents.

**Table 1:** Sequence homologies of target genes. Given are the pairwise homologies of target genes in % based on cDNA sequence

	AtLNG1	AtSEX1	AtNPQ1	AtMAX3	AtHY2	AtGUN4
AtSEX1	38.06					
AtNPQ1	19.02	15.42				
AtMAX3	21.01	16.34	39.25			
AtHY2	14.70	11.88	26.32	25.55		
AtGUN4	10.20	8.25	19.58	18.67	21.36	
AtTRY	7.31	5.47	12.95	13.79	16.93	20.61

**Fig. 2:**
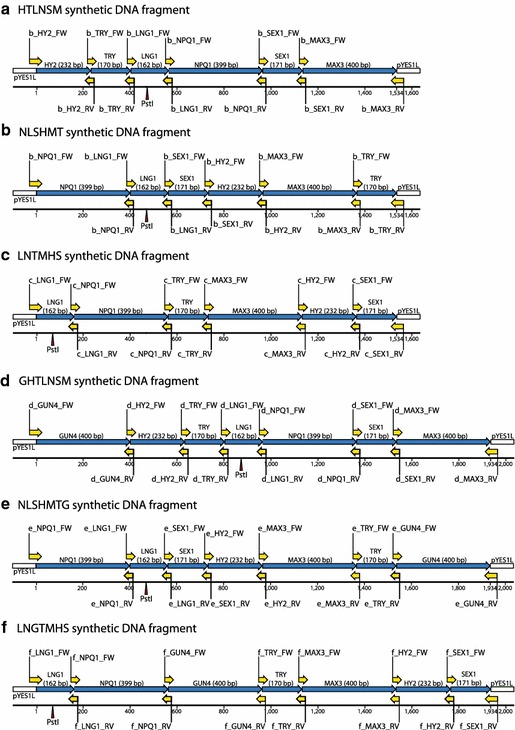
Synthetic multiple RNAi fragments assembled in pYES1L from double-stranded PCR products by a single yeast transformation event. Primers used to amplify individual gene specific tags (GSTs) are given in *yellow*. Synthetic fragments (**a**–**c**) contain six different GSTs in different orders, while fragments (**d**–**f**) carry an additional GST for the gene GUN4. Total fragment lengths are 1534 bp for the six-gene and 1934 bp for the seven-gene multiple RNAi fragment. *Pst*I restriction sites are labeled by *red arrows*

**Fig. 3:**
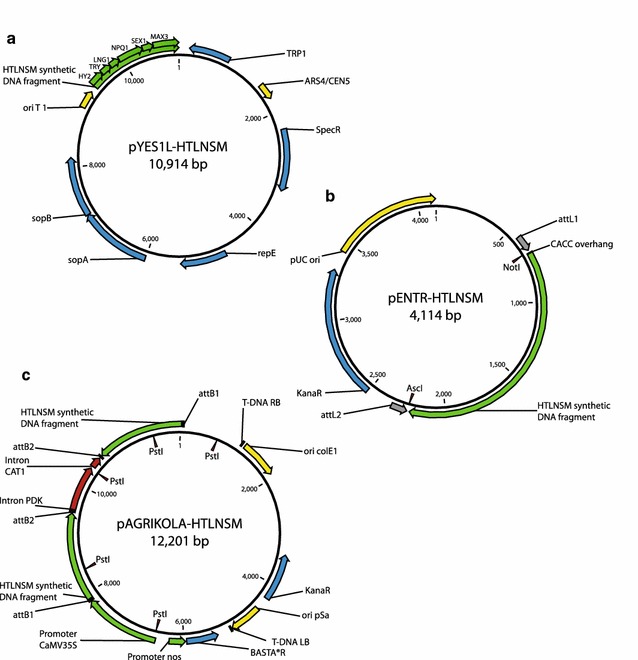
Series of vectors created to clone the HTLNSM synthetic multiple RNAi fragment. **a** Gene specific tags for *AtHY2*, *AtTRY*, *AtLNG1*, *AtNPQ1*, *AtSEX1* and *AtMAX3* were assembled to form the HTLNSM multiple RNAi fragment in yeast. **b** The HTLNSM synthetic DNA fragment was amplified by PCR using pYES1L-HTLNSM as template and the PCR product was cloned into pENTR™/D-TOPO to create pENTR-HTLNSM. The CACC-overhang was introduced to facilitate directional cloning into pENTR™/D-TOPO. **c** The HTLNSM synthetic DNA fragment was subsequently transferred to pAGRIKOLA by LR recombination reaction to create the hpRNAi vector pAGRIKOLA-HTLNSM. Refer to Thermo Fisher Scientific Inc., (www.thermofisher.com) for more information about pYES1L and pENTRY and to Hilson et al. [20] for pAGRIKOLA

The CATMA database [47] provides GST sequences for *A. thaliana* genes that were developed using specific primer and amplicon design software [48] and undergo certain quality controls. In favor of standardization, we decided to make use of this resource. GSTs for the selected target genes *AtHY2* (H), *AtTRY* (T), *AtLNG1* (L), *AtNPQ1* (N), *AtSEX1* (S), *AtMAX3* (M) and *AtGUN4* (G), were amplified and assembled in silico to compose synthetic RNAi fragments consisting of either six or seven GSTs in alternate orders (Fig. 2). The order of the GSTs in the seven GST fragment was chosen randomly. The six GST fragments followed the same order without including the AtGUN4-GST. Six different multiple RNAi synthetic DNA fragments were designed: (a) HTLNSM, (b) NLSHMT, (c) LNTMHS, (d) GHTLNSM, (e) NLSHMTG and (f) LNGTMHS. Oligonucleotide primers to amplify the individual GSTs from an *Arabidopsis* cDNA library that contain a 5′ extension of DNA homologous to the neighboring GST in the respective synthetic DNA fragment were designed using a web based tool (http://www.thermofisher.com/order/oligoDesigner). Individual GSTs were PCR amplified and multiple RNAi synthetic DNA fragments were assembled in yeast using plasmid pYES1L as a backbone (Figs. 1, 2, 3). The assembled pYES1L plasmids served as template to PCR amplify the synthetic DNA fragments and to create respective pENTR clones (Figs. 1, 3). The cloned synthetic DNA fragments were subsequently transferred to the binary plant hpRNA vector pAGRIKOLA [20] by Gateway^®^ Cloning [49]. The entire cloning of the multiple RNAi pAGRIKOLA vectors is cost and time-effective compared to other cloning strategies (e.g., use of restriction endonucleases, ligases and multiple subcloning steps) and can be completed within 2 weeks for any chosen GST combination (Fig. 1). Even though we used Gateway^®^ Technology for cloning, since the assembly process itself is independent of any restriction sites, final cloning of the assembled fragment can easily be adapted to restriction enzyme based protocols, if respective target plasmids are available. After transformation of *Agrobacterium tumefaciens* with the pAGRIKOLA vectors, *A. thaliana* Col-0 plants were transformed and T1 transgenic lines were selected.

### RNAi-mediated knockdown of single target genes

**Fig. 4:**
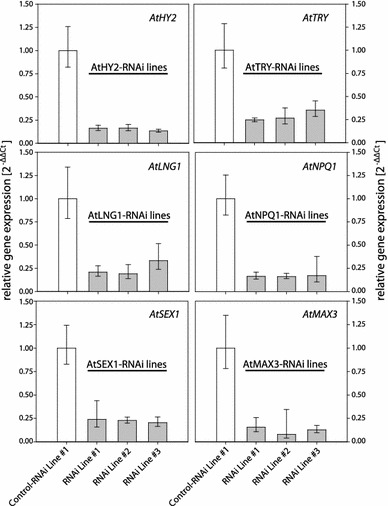
RNAi mediated down-regulation of target gene expression in single *Arabidopsis* hpRNAi lines. T2 individuals of three individual pAGRIKOLA hpRNAi expressing *Arabidopsis* lines were tested for each of the target genes, *AtHY2*, *AtTRY*, *AtLNG1*, *AtNPQ1*, *AtSEX1* and *AtMAX3*. Abundance of the respective target transcript are given as relative expression $(2^{{ - \Delta \Delta {\text{C}}_{\text{T}} }} )$ compared to that of an *Arabidopsis* line expressing a pAGRIKOLA-Control RNAi fragment and *AtACT2* for normalization

In order to test whether the chosen single/individual GSTs can efficiently silence the target gene expression, *Arabidopsis* plants were transformed with respective single GST pAGRIKOLA vectors and the transcript abundance in individual RNAi lines was determined by qRT-PCR (Fig. 4). We successfully obtained *Arabidopsis* RNAi lines for the target genes *AtHY2*, *AtTRY*, *AtLNG1*, *AtNPQ1*, *AtSEX1* and *AtMAX3* and target transcript abundance declined to 20–25 % of the control (Fig. 4). RNAi-mediated down-regulation of the six target genes did not result in a visible phenotype as previously described for the respective loss of function mutants phenotype [50–55], implying that the observed 80 % reduction in transcript levels is not sufficient to cause a decrease of cellular target protein amounts as seen in these mutants. Nonetheless, we showed here that with exception of AtGUN4 the selected genes could be knocked down via the RNAi approach.

Alternatively, even with several rounds of transformation we were unable to select *AtGUN4* RNAi lines. This is in contrast to the work of Du et al. [56] who successfully transformed *Arabidopsis* with a *AtGUN4* RNAi plasmid and Schwab et al. [57], who efficiently down-regulated *AtGUN4* expression using a microRNA approach. Since the two mentioned approaches used the *A. tumefaciens* mannopine synthase promoter or a set of tissue specific promoters, and we used a pAGRIKOLA that contains the CaMV 35S promoter driving the RNAi expression, we speculated that different promoter activities may have caused the failure to select *AtGUN4* RNAi transgenic lines. We cannot rule out the possibility that we were unable to distinguish severe *AtGUN4* RNAi effects and BASTA^®^ sensitivity of Arabidopsis seedlings during T1 selection since both of them would result in retarded growth and yellow seedlings. The originally described *Arabidopsisgun4*-*1* mutant’s yellow leaf phenotype is caused by a missense mutation resulting in a leucine to phenylalanine exchange at position 88 of the 265 amino acid sequence [58], indicating that key changes in AtGUN4 protein result in a drastic phenotype. However, since we screened hundreds of thousands of T1 seeds for the *AtGUN4*-GST containing seven-gene RNAi fragment and eventually were able to obtain two multiple-RNAi transformants where expression of seven genes including *AtGUN4* was down regulated (described below), we did not omit *AtGUN4* as a target gene for our proof-of-concept study.

We also created *Arabidopsis* RNAi control lines that were transformed with the pAGRIKOLA-control vector and expressed an untargeted hpRNAi construct. These lines were used as standard comparators for quantitative expression analysis, as they can be treated with the herbicide BASTA^®^ similar to the multiple RNAi lines. Moreover, it has been shown that the expression of RNAi constructs itself can result in unwanted and off-target effects on nuclear gene expression [59] and comparing gene expression of wild-type and RNAi plants can give false positive results.

### Efficient silencing of multiple transcripts by synthetic hpRNA fragments

**Fig. 5:**
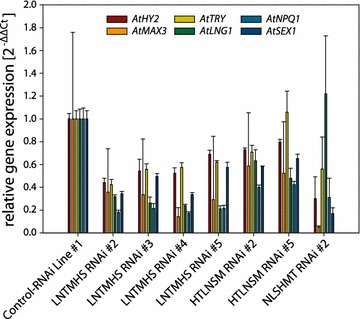
Simultaneous RNAi mediated down-regulation of expression of six target genes in flowers of *Arabidopsis* hpRNAi lines transformed with a single RNAi construct. Four *Arabidopsis* T2 lines transformed with pAGRIKOLA-LNTMHS, two T2 lines transformed with pAGRIKOLA-HTLNSM, and one T2 line transformed with pAGRIKOLA-NLSHMT were tested for each of the target genes, *AtHY2*, *AtTRY*, *AtLNG1*, *AtNPQ1*, *AtSEX1* and *AtMAX3*. Abundance of the respective target transcript are given as relative expression $(2^{{ - \Delta \Delta {\text{C}}_{\text{T}} }} )$ compared to that of an *Arabidopsis* line expressing a pAGRIKOLA-Control RNAi fragment and *AtACT2* for normalization

After transformation of *Arabidopsis* with six GST constructs, we selected 10 individual lines for each of the pAGRIKOLA-LNTMHS, the pAGRIKOLA-HTLNSM, and the pAGRIKOLA-NLSHMT constructs. Figure 5 summarizes the expression of target genes as monitored in T2 individuals of four lines transformed with pAGRIKOLA-LNTMHS, two lines transformed with pAGRIKOLA-HTLNSM, and one line transformed with pAGRIKOLA-NLSHMT. Expression of target genes in leaf and flower tissue was successfully downregulated in all lines, with the exception of *AtTRY* in HTLNSM RNAi line #5 and *AtLNG1* in NLSHMT RNAi line #2 (Fig. 5). Level of downregulation ranged between 20 and 90 % compared to the *Arabidopsis* Control-RNAi line. Comparison of data obtained with the three constructs carrying the GSTs in different order reveals no obvious differences in efficiency of the RNAi effect on the respective target gene (Fig. 5).

**Fig. 6:**
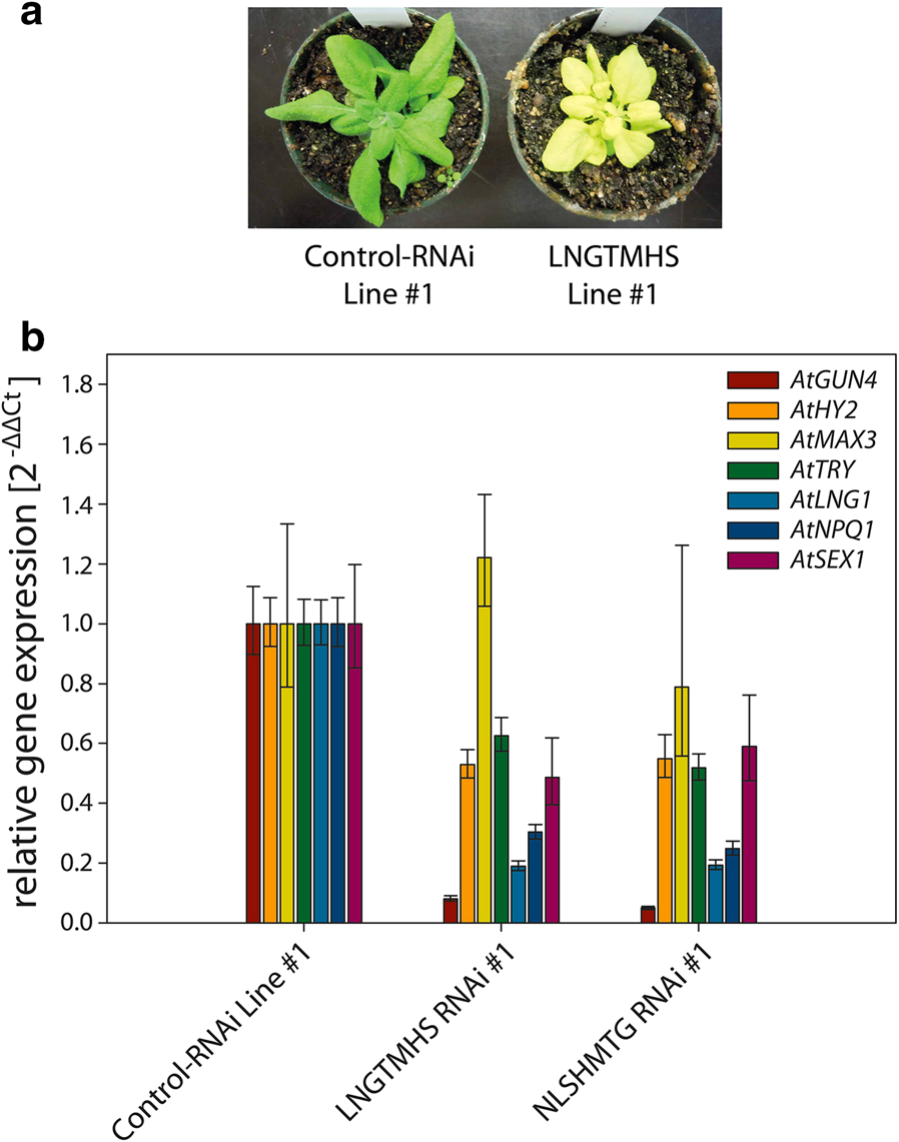
Simultaneous RNAi mediated down-regulation of expression of seven target genes in *Arabidopsis* hpRNAi lines transformed with a single RNAi construct. **a** Phenotype of one T1 individual of *Arabidopsis* transformed with pAGRIKOLA-LNGTMHS. **b** Leaves of one T1 individual of *Arabidopsis* transformed with pAGRIKOLA-LNGTMHS, and one T1 individual transformed with pAGRIKOLA-NLSHMTG were tested for expression of each of the target genes, *AtGUN4*, *AtHY2*, *AtTRY*, *AtLNG1*, *AtNPQ1*, *AtSEX1* and *AtMAX3*. Abundance of the respective target transcripts are given as relative expression $(2^{{ - \Delta \Delta {\text{C}}_{\text{T}} }} )$ compared to that of an *Arabidopsis* line expressing a pAGRIKOLA-Control RNAi fragment and *AtACT2* for normalization

For each of the constructs containing seven GSTs pAGRIKOLA-NLSHMTG and pAGRIKOLA-LNGTMHS, only one successful *Arabidopsis* transformant could be selected. We were unable to select a line expressing the GHTLNSM construct and we can only speculate that the low transformation efficiency is due to lethal downregulation of *AtGUN4* expression as mentioned above. Both of the seven GST lines show the similar chlorotic phenotype and had only about 10 % of the *GUN4* control transcript level remaining (Fig. 6a, b), indicating disturbed chlorophyll biosynthesis [58, 60, 61]. However, Fig. 6b shows the effects of the two seven-GST RNAi constructs on the expression of the respective target genes. Similar to the results with six individual GST constructs, with the exception of *AtMAX3*, the expression of each of the target genes is downregulated by 40 % (AtLNG1, AtSEX1) to 90 % (AtGUN4).

## Conclusions

We present a novel and cost-effective strategy to simultaneously down regulate expression of more than one target gene in plants using a single synthetic construct. Similar to gene stacking, where expression of different transgenes is triggered by a single construct, this method circumvents issues with introducing more than one transformation marker, characterizing multiple T-DNA insertion sites or laborious crossing of different mutant lines. Using the yeast assembly system reduces the cloning work and can quickly be established. We do not conceal that selecting a transformation event or transformed line that shows sufficient knock down of all targeted genes may result in screening of hundreds of individuals. Assuming that typically about one out of five *Arabidopsis* RNAi lines show sufficient down regulation of a single target gene, it might be necessary to screen about 5^n^ individual transformants when n genes are targeted with our suggested approach. Comparison of RNAi efficiencies obtained with single or multiple RNAi constructs (Figs. 4, 5, 6) may lead to the conclusion that the multiple RNAi constructs act less efficiently. We cannot rule out such an interfering mechanism but also want to present a hypothesis that this might be an apparent effect based on the relatively low amount of individuals expressing the multiple RNAi constructs. In case of the single RNAi plants we were able to choose the best three out of approx. 15–20 selected transformants for each construct. We would roughly expect that screening of 15,625 (5^6^) or even 78,125 (5^7^) selected individuals would be necessary to identify mutants with similar levels of downregulation for all targeted genes. For our proof-of-concept study, we rather intended to provide evidence that a single synthetic RNAi fragment can affect a large panel of transcripts. In practical approaches, where e.g., up to four genes are targeted, only about 625 transformants would need to be screened for desired transgenic lines. However, the increasing amount of individual RNAi plants that need to be screened for best downregulation result is likely independent of our approach and also true for other ways to address multiple target genes (e.g., multiple transformations or serial cloning of constructs). Applying the presented method to artificial micro RNA (amiRNA) expression instead of hpRNAi, may solve this problem, because amiRNA are even more effective in target gene down regulation [57, 62, 63]. Multiple gene knockdown in plants using a single synthetic construct may facilitate ambitious projects dealing with establishing new biochemical pathways, e.g., transition from C3 to C4 photosynthesis [64, 65] and/or from C3 to CAM photosynthesis [66, 67], especially for rewiring the diel expression pattern of genes shared between C3 and CAM species.

## Methods

### Experimental design

Based on the work of Lloyd and Meinke [46], who provide an excellent overview about *Arabidopsis* phenotypes caused by disrupted genes, we chose six and/or seven genes that can likely be knocked down simultaneously in a single *Arabidopsis* plant without generating a lethal phenotype; they were: *AtHY2* (At3g09150) [50], *AtLNG1* (At5g15580) [51], *AtTRY* (At5g53200) [52], *AtMAX3* (At2g44990) [53], *AtNPQ1* (At1g08550) [54], *AtSEX1* (At1g10760) [55] and *AtGUN4* (At3g59400) [58]. The CATMA database [47] was used to identify specific gene sequence tags (GST) and respective cloning primer sequences for each of the seven transcripts (Additional file 2: Table S1). In order to test the functionality of the GSTs for RNAi in *Arabidopsis*, single GSTs were cloned and inserted into the binary plant hpRNAi plasmid pAGRIKOLA [20]. The expression of the resulting hpRNA-constructs contained the respective GSTs that were cloned in sense and antisense orientation flanking the *Pdk* and *Cat* introns was driven by the CaMV 35S promoter. *A. thaliana* ‘Columbia-0’ (Col-0) plants were transformed with these vectors and transcript levels of the respective target genes were determined in herbicide resistant T2 individuals. The same GSTs were used to assemble synthetic RNAi fragments. Three different synthetic RNAi fragments were designed for two gene sets (six or seven gene knockdown), comprising varying assembly orders to evaluate sequence effects on RNAi effectiveness. We randomly chose the order of the GSTs for the seven GST fragments. The six GST fragments followed the same order without assembling the AtGUN4-GST. This strategy enabled multiple use of assembly primers (Additional file 2: Table S1). The synthetic RNAi fragments were eventually cloned into pAGRIKOLA and after *Arabidopsis* transformation, transcript levels of the target genes were determined in T1 or T2 individuals selected for presence of the transgene by BASTA^®^ treatment. In all experiments, *Arabidopsis* plants transformed with pAGRIKOLA carrying no GST were used as controls.

### Cloning of single RNAi plasmids and controls

Single GSTs were amplified from an *A. thaliana* Col-0 cDNA template using Phusion High-Fidelity DNA Polymerase (Thermo Fisher Scientific Inc.,) and primers listed in Additional file 2: Table S1 according to the manufacturer’s protocols. 5′-CACC-3′ overhangs were introduced with the PCR primers to facilitate directional cloning into pENTR™/D-TOPO (Life Technologies). Gel purified PCR products were subcloned into pENTR™/D-TOPO and resulting pENTR clones were verified by sequencing. Since pENTR and the destination plasmid pAGRIKOLA carry the same bacterial selection marker (*nptI*), pENTR plasmids were serially digested with *PvuI* and *NruI* and the linearized plasmid backbones were gel purified before LR clonase II recombination reactions (Thermo Fisher Scientific Inc.,) and were performed to create the pAGRIKOLA RNAi expression plasmids. The correct insertions of the GSTs into the resulting pAGRIKOLA RNAi vectors were verified by sequencing using primers listed in Additional file 2: Table S1.

An empty plasmid control was created by cloning a pENTR-control plasmid transferring the sequence 5′-CACCAAAATG-3′ into the respective destination plasmid pAGRIKOLA. Direct subcloning of the very short linker sequence into pENTR™/D-TOPO by means of annealed oligos was not successful. Therefore, one of the pENTR clones (pENTR-AtHY2) was chosen, digested with *AscI* and *NotI* and gel purified to release the inserted AtHY2 GST. Two synthetic oligos (5′-GGCCGCCCCCTTCACCAAAATGAAGGGTGGG-3′, 5′-CGCGCCCACCCTTCATTTTGGTGAAGGGGGC-3′) were annealed to create a linker by heating 1 nmol of each oligo for 5 min at 95 °C in 100 µl buffer (10 mM Tris-HCl pH 7.5, 10 mM MgCl_2_) and slowly cooling down at a rate of 2 °C per min. Ten pmol of the linker were subsequently ligated into 100 nmol linearized pENTR using T4 DNA Ligase (Thermo Fisher Scientific Inc.,) according to the manufacturer’s protocol. Competent *E. coli* (strain NEB 5-alpha, New England Biolabs) were transformed with the ligation mixture and a correctly assembled pENTR-control plasmid was identified by restriction digestion and sequencing. An LR clonase II recombination reaction to create a pAGRIKOLA-control vector and subsequent sequencing were performed as described above.

### Assembly of multiple RNAi constructs

The GeneArt^®^ High-Order Genetic Assembly Kit and the GeneArt^®^ pYES1L Vector with Sapphire™ Technology (Thermo Fisher Scientific Inc.,) were used to assemble the multiple RNAi constructs in yeast. Overlapping primers to amplify GSTs were designed using the GeneArt^®^ Primer and Construct Design Tool (http://www.thermofisher.com/order/oligoDesigner). Single GSTs to be assembled to multiple RNAi fragments were amplified from an *A. thaliana* Col-0 cDNA template using Phusion High-Fidelity DNA Polymerase and primers listed in Table S1 according to the manufacturer’s protocols. PCR products were gel purified, concentrations were determined and respective PCR products and pYES1L were mixed and chemical competent yeast cells were transformed according to the manufacturer’s protocol in order to create (a) pYES1L-HTLNSM, (b) pYES1L-NLSHMT, (c) pYES1L-LNTMHS, (d) pYES1L-GHTLNSM, (e) pYES1L-NLSHMTG and (f) pYES1L-LNGTMHS (Figs. 2, 3). After 3 days incubation on yeast selection medium, eight yeast colonies were picked for each assembly, plasmid DNAs were extracted according to the The GeneArt^®^ High-Order Genetic Assembly Kit protocol and used as template for PCR proof of successful assembly using primers given in Additional file 2: Table S1. One plasmid preparation for a given PCR product of the expected size was used as template for PCR for a subsequent cloning of the assembled synthetic fragments into pENTR™/D-TOPO. Full length sequences of the assembled fragments were obtained from the respective pENTR clones.

### Cloning of multiple RNAi plasmids

One yeast plasmid preparation for each assembled RNAi fragment creating a PCR product of the expected size was used as template for PCR for a subsequent cloning of the assembled synthetic fragments into pENTR™/D-TOPO. Therefore, synthetic RNAi fragments were amplified using Phusion High-Fidelity DNA Polymerase and primers listed in Additional file 2: Table S1. 5′-CACC-3′ overhangs were introduced with the PCR primers to facilitate directional cloning into pENTR™/D-TOPO. Gel purified PCR products were subcloned into pENTR™/D-TOPO and resulting pENTR clones were verified by sequencing. For transferring the cloned synthetic fragments into pAGRIKOLA, pENTR plasmids were serially digested with *PvuI* and *NruI* and LR clonase II recombination reactions were performed as described above. The correct insertions of the synthetic RNAi fragments into the resulting pAGRIKOLA multiple RNAi vectors were verified by restriction digestions using *PstI* and sequencing using primers listed in Additional file 2: Table S1.

### Plant growth conditions and plant transformation

Seeds of *A. thaliana* Col-0 were directly sown into soil and seedlings were transplanted to single pots after 2 weeks. Seedlings and plants were grown in growth chamber at 23 °C and approximately 200 µE light in long-day conditions (14 h of light).

Chemically competent cells [68] of *A. tumefaciens* strain GV3101(pMP90) [69] were prepared and transformed with the helper plasmid pSOUP [70]. A resulting GV3101::pMP90::pSOUP clone was again made chemically competent and transformed with the single RNAi and the multiple RNAi pAGRIKOLA vectors. *Agrobacteria* clones were used to transform *Arabidopsis* plants by floral dip [71]. Transformed T1 seedlings were selected by spraying 2-week-old soil-grown seedlings with 0.01 % (w/v) BASTA^®^ five times in 2 days intervals. T1 seeds transformed with RNAi fragments containing the *AtGUN4* GST were surface sterilized by serial washing with 96 % (v/v) ethanol, 20 % (v/v) household bleach supplemented with 0.05 % (v/v) Tween-20, and water, and placed at 4 °C for 2 days. Seeds were subsequently plated on ½ Murashige and Skoog (MS) medium [72] supplemented with 1 % (w/v) sucrose, 0.01 % (w/v) BASTA^®^ and 0.8 % (w/v) agar, and germinated under continuous light at approximately 90 µmol photons m^−2^ s^−1^. Successfully selected lines were transplanted and grown until mature T2 seeds could be harvested. T2 seeds of pAGRIKOLA-RNAi lines, as well as pAGRIKOLA-control lines, were grown on soil and sprayed with BASTA^®^ to remove segregating wild-type individuals.

### RNA extraction and gene expression analysis

Total RNA was extracted from leaves of 10 days and 4-week-old seedlings or flowers of 12-week-old plants, respectively using the Invisorb Spin Plant Mini Kit (Stratec Molecular). Two µg of total RNA were reversely transcribed in cDNA using an Oligo-dT_18_ primer and Thermo Scientific RevertAid Reverse Transcriptase (Thermo Fisher Scientific Inc.,). Quantitative RT-PCR (qRT) was conducted to examine the transcript levels of each target gene using a StepOnePlus (Applied Biosystems), Maxima SYBR Green/ROX qPCR Master Mix (Thermo Scientific) and cDNA corresponding to 80 ng RNA in a total volume of 25 µl. Three biological replicates were used for each qRT analysis. The following cycling conditions were applied for PCR: 10 min at 95 °C, 35 cycles of 15 s at 95 °C and 60 s at 60 °C. Transcript level was normalized against *AtACTIN2* (*At3g18780.1*) and an *Arabidopsis* Col-0 pAGRIKOLA-control line and are presented as $2^{{ - \Delta \Delta {\text{C}}_{\text{T}} }}$ values [73, 74]. Gene-specific qRT primers were designed using QuantPrime [75]. All primers used for qRT analysis are listed in Additional file 2: Table S1.

## Additional files

10.1186/s12014-016-9104-2 Alignment of cDNAs of *AtHY2*, *AtTRY*, *AtLNG1*, *AtNPQ1*, *AtSEX1*, *AtMAX3* and *AtGUN4*.
10.1186/s12014-016-9104-2 PCR primers used in this study.

## Abbreviations

amiRNA: artificial micro RNA
dsRNA: double-stranded RNA
GST: gene specific tag
hpRNA: hairpin RNA
PTGS: post transcriptional gene silencing
qRT: quantitative RT-PCR
RNAi: RNA interference
RISC: RNA-induced silencing complex
shRNA: small hairpin RNA
siRNA: small interfering RNA
ssRNA: single-stranded RNA
TAR: transformation associated recombination

## References

[1] Fire A, Xu S, Montgomery MK, Kostas SA, Driver SE, Mello CC (1998). Potent and specific genetic interference by double-stranded RNA in *Caenorhabditis elegans*. Nature.

[2] Wilson RC, Doudna JA (2013). Molecular mechanisms of RNA interference. Annu Rev Biophys.

[3] Agrawal N, Dasaradhi PV, Mohmmed A, Malhotra P, Bhatnagar RK, Mukherjee SK (2003). RNA interference: biology, mechanism, and applications. Microbiol Mol Biol Rev.

[4] Hannon GJ (2002). RNA interference. Nature.

[5] Herr AJ, Baulcombe DC (2004). RNA silencing pathways in plants. Cold Spring Harb Symp Quant Biol.

[6] Baulcombe D (2004). RNA silencing in plants. Nature.

[7] Watanabe T, Totoki Y, Toyoda A, Kaneda M, Kuramochi-Miyagawa S, Obata Y, Chiba H, Kohara Y, Kono T, Nakano T (2008). Endogenous siRNAs from naturally formed dsRNAs regulate transcripts in mouse oocytes. Nature.

[8] Buchon N, Vaury C (2006). RNAi: a defensive RNA-silencing against viruses and transposable elements. Heredity.

[9] Ito H (2013). Small RNAs and regulation of transposons in plants. Genes Genet Syst.

[10] Dunoyer P, Brosnan CA, Schott G, Wang Y, Jay F, Alioua A, Himber C, Voinnet O (2010). An endogenous, systemic RNAi pathway in plants. EMBO J.

[11] Okamura K, Lai EC (2008). Endogenous small interfering RNAs in animals. Nat Rev Mol Cell Biol.

[12] Ding SW, Voinnet O (2007). Antiviral immunity directed by small RNAs. Cell.

[13] Obbard DJ, Gordon KH, Buck AH, Jiggins FM (2009). The evolution of RNAi as a defence against viruses and transposable elements. Philos Trans R Soc Lond B Biol Sci.

[14] Senthil-Kumar M, Mysore K, Catalano A (2010). RNAi in plants: recent developments and applications in agriculture. Gene silencing: theory, techniques and applications.

[15] Gilchrist E, Haughn G (2010). Reverse genetics techniques: engineering loss and gain of gene function in plants. Brief Funct Genomics.

[16] Watson JM, Fusaro AF, Wang M, Waterhouse PM (2005). RNA silencing platforms in plants. FEBS Lett.

[17] Eamens A, Wang MB, Smith NA, Waterhouse PM (2008). RNA silencing in plants: yesterday, today, and tomorrow. Plant Physiol.

[18] Fusaro AF, Matthew L, Smith NA, Curtin SJ, Dedic-Hagan J, Ellacott GA, Watson JM, Wang MB, Brosnan C, Carroll BJ (2006). RNA interference-inducing hairpin RNAs in plants act through the viral defence pathway. EMBO Rep.

[19] Wesley SV, Helliwell CA, Smith NA, Wang MB, Rouse DT, Liu Q, Gooding PS, Singh SP, Abbott D, Stoutjesdijk PA (2001). Construct design for efficient, effective and high-throughput gene silencing in plants. Plant J.

[20] Hilson P, Allemeersch J, Altmann T, Aubourg S, Avon A, Beynon J, Bhalerao RP, Bitton F, Caboche M, Cannoot B (2004). Versatile gene-specific sequence tags for Arabidopsis functional genomics: transcript profiling and reverse genetics applications. Genome Res.

[21] Wielopolska A, Townley H, Moore I, Waterhouse P, Helliwell C (2005). A high-throughput inducible RNAi vector for plants. Plant Biotechnol J.

[22] Gendler K, Paulsen T, Napoli C (2008). ChromDB: the chromatin database. Nucleic Acids Res.

[23] Halpin C (2005). Gene stacking in transgenic plants—the challenge for 21st century plant biotechnology. Plant Biotechnol J.

[24] Naqvi S, Farre G, Sanahuja G, Capell T, Zhu C, Christou P (2010). When more is better: multigene engineering in plants. Trends Plant Sci.

[25] Ye X, Al-Babili S, Kloti A, Zhang J, Lucca P, Beyer P, Potrykus I (2000). Engineering the provitamin A (beta-carotene) biosynthetic pathway into (carotenoid-free) rice endosperm. Science.

[26] Buntru M, Gartner S, Staib L, Kreuzaler F, Schlaich N (2013). Delivery of multiple transgenes to plant cells by an improved version of MultiRound Gateway technology. Transgenic Res.

[27] Wang Y, Yau YY, Perkins-Balding D, Thomson JG (2011). Recombinase technology: applications and possibilities. Plant Cell Rep.

[28] Vemanna RS, Chandrashekar BK, Hanumantha Rao HM, Sathyanarayanagupta SK, Sarangi KS, Nataraja KN, Udayakumar M (2013). A modified MultiSite gateway cloning strategy for consolidation of genes in plants. Mol Biotechnol.

[29] Sun Q, Liu J, Li Y, Zhang Q, Shan S, Li X, Qi B (2013). Creation and validation of a widely applicable multiple gene transfer vector system for stable transformation in plant. Plant Mol Biol.

[30] Motegi Y, Katayama K, Sakurai F, Kato T, Yamaguchi T, Matsui H, Takahashi M, Kawabata K, Mizuguchi H (2011). An effective gene-knockdown using multiple shRNA-expressing adenovirus vectors. J Control Release.

[31] Xu XM, Yoo MH, Carlson BA, Gladyshev VN, Hatfield DL (2009). Simultaneous knockdown of the expression of two genes using multiple shRNAs and subsequent knock-in of their expression. Nat Protoc.

[32] Chumakov SP, Kravchenko JE, Prassolov VS, Frolova EI, Chumakov PM (2010). Efficient downregulation of multiple mRNA targets with a single shRNA-expressing lentiviral vector. Plasmid.

[33] Zhu X, Santat LA, Chang MS, Liu J, Zavzavadjian JR, Wall EA, Kivork C, Simon MI, Fraser ID (2007). A versatile approach to multiple gene RNA interference using microRNA-based short hairpin RNAs. BMC Mol Biol.

[34] Sun D, Melegari M, Sridhar S, Rogler CE, Zhu L (2006). Multi-miRNA hairpin method that improves gene knockdown efficiency and provides linked multi-gene knockdown. Biotechniques.

[35] Sander JD, Joung JK (2014). CRISPR–Cas systems for editing, regulating and targeting genomes. Nat Biotechnol.

[36] Piatek A, Ali Z, Baazim H, Li L, Abulfaraj A, Al-Shareef S, Aouida M, Mahfouz MM (2015). RNA-guided transcriptional regulation in planta via synthetic dCas9-based transcription factors. Plant Biotechnol J.

[37] Feng Z, Zhang B, Ding W, Liu X, Yang DL, Wei P, Cao F, Zhu S, Zhang F, Mao Y (2013). Efficient genome editing in plants using a CRISPR/Cas system. Cell Res.

[38] Xie K, Yang Y (2013). RNA-guided genome editing in plants using a CRISPR–Cas system. Mol Plant.

[39] Larionov V, Kouprina N, Graves J, Chen XN, Korenberg JR, Resnick MA (1996). Specific cloning of human DNA as yeast artificial chromosomes by transformation-associated recombination. Proc Natl Acad Sci USA.

[40] Gibson DG (2009). Synthesis of DNA fragments in yeast by one-step assembly of overlapping oligonucleotides. Nucleic Acids Res.

[41] Orr-Weaver TL, Szostak JW, Rothstein RJ (1981). Yeast transformation: a model system for the study of recombination. Proc Natl Acad Sci USA.

[42] Gibson DG, Benders GA, Axelrod KC, Zaveri J, Algire MA, Moodie M, Montague MG, Venter JC, Smith HO, Hutchison CA (2008). One-step assembly in yeast of 25 overlapping DNA fragments to form a complete synthetic *Mycoplasma genitalium* genome. Proc Natl Acad Sci USA.

[43] Gibson DG, Benders GA, Andrews-Pfannkoch C, Denisova EA, Baden-Tillson H, Zaveri J, Stockwell TB, Brownley A, Thomas DW, Algire MA (2008). Complete chemical synthesis, assembly, and cloning of a *Mycoplasma genitalium* genome. Science.

[44] Bigger BW, Liao AY, Sergijenko A, Coutelle C (2011). Trial and error: how the unclonable human mitochondrial genome was cloned in yeast. Pharm Res.

[45] Raymond CK, Sims EH, Olson MV (2002). Linker-mediated recombinational subcloning of large DNA fragments using yeast. Genome Res.

[46] Lloyd J, Meinke D (2012). A comprehensive dataset of genes with a loss-of-function mutant phenotype in Arabidopsis. Plant Physiol.

[47] Crowe ML, Serizet C, Thareau V, Aubourg S, Rouze P, Hilson P, Beynon J, Weisbeek P, van Hummelen P, Reymond P (2003). CATMA: a complete Arabidopsis GST database. Nucleic Acids Res.

[48] Thareau V, Dehais P, Serizet C, Hilson P, Rouze P, Aubourg S (2003). Automatic design of gene-specific sequence tags for genome-wide functional studies. Bioinformatics.

[49] Hartley JL, Temple GF, Brasch MA (2000). DNA cloning using in vitro site-specific recombination. Genome Res.

[50] Koornneef M, Rolff E, Spruit CJP (1980). Genetic control of light-inhibited hypocotyl elongation in *Arabidopsis thaliana* (L.) Heynh. Z Pflanzenphysiol.

[51] Lee YK, Kim GT, Kim IJ, Park J, Kwak SS, Choi G, Chung WI (2006). LONGIFOLIA1 and LONGIFOLIA2, two homologous genes, regulate longitudinal cell elongation in Arabidopsis. Development.

[52] Hulskamp M, Misra S, Jurgens G (1994). Genetic dissection of trichome cell development in Arabidopsis. Cell.

[53] Booker J, Auldridge M, Wills S, McCarty D, Klee H, Leyser O (2004). MAX3/CCD7 is a carotenoid cleavage dioxygenase required for the synthesis of a novel plant signaling molecule. Curr Biol.

[54] Niyogi KK, Grossman AR, Bjorkman O (1998). Arabidopsis mutants define a central role for the xanthophyll cycle in the regulation of photosynthetic energy conversion. Plant Cell.

[55] Yu TS, Kofler H, Hausler RE, Hille D, Flugge UI, Zeeman SC, Smith AM, Kossmann J, Lloyd J, Ritte G (2001). The Arabidopsis sex1 mutant is defective in the R1 protein, a general regulator of starch degradation in plants, and not in the chloroplast hexose transporter. Plant Cell.

[56] Du SY, Zhang XF, Lu Z, Xin Q, Wu Z, Jiang T, Lu Y, Wang XF, Zhang DP (2012). Roles of the different components of magnesium chelatase in abscisic acid signal transduction. Plant Mol Biol.

[57] Schwab R, Ossowski S, Riester M, Warthmann N, Weigel D (2006). Highly specific gene silencing by artificial microRNAs in Arabidopsis. Plant Cell.

[58] Larkin RM, Alonso JM, Ecker JR, Chory J (2003). GUN4, a regulator of chlorophyll synthesis and intracellular signaling. Science.

[59] Schlicke H, Hartwig AS, Firtzlaff V, Richter AS, Glasser C, Maier K, Finkemeier I, Grimm B (2014). Induced deactivation of genes encoding chlorophyll biosynthesis enzymes disentangles tetrapyrrole-mediated retrograde signaling. Mol Plant.

[60] Wilde A, Mikolajczyk S, Alawady A, Lokstein H, Grimm B (2004). The gun4 gene is essential for cyanobacterial porphyrin metabolism. FEBS Lett.

[61] Peter E, Grimm B (2009). GUN4 is required for posttranslational control of plant tetrapyrrole biosynthesis. Mol Plant.

[62] Tiwari M, Sharma D, Trivedi PK (2014). Artificial microRNA mediated gene silencing in plants: progress and perspectives. Plant Mol Biol.

[63] Ossowski S, Schwab R, Weigel D (2008). Gene silencing in plants using artificial microRNAs and other small RNAs. Plant J.

[64] Covshoff S, Hibberd JM (2012). Integrating C4 photosynthesis into C3 crops to increase yield potential. Curr Opin Biotechnol.

[65] von Caemmerer S, Quick WP, Furbank RT (2012). The development of C4 rice: current progress and future challenges. Science.

[66] Yang X, Cushman JC, Borland AM, Edwards EJ, Wullschleger SD, Tuskan GA, Owen NA, Griffiths H, Smith JA, De Paoli HC (2015). A roadmap for research on crassulacean acid metabolism (CAM) to enhance sustainable food and bioenergy production in a hotter, drier world. New Phytol.

[67] DePaoli HC, Borland AM, Tuskan GA, Cushman JC, Yang X (2014). Synthetic biology as it relates to CAM photosynthesis: challenges and opportunities. J Exp Bot.

[68] Höfgen R, Willmitzer L (1988). Storage of competent cells for Agrobacterium transformation. Nucleic Acids Res.

[69] Koncz C, Schell J (1986). The promoter of TL-DNA gene 5 controls the tissue-specific expression of chimaeric genes carried by a novel type of Agrobacterium binary vector. Mol Gen Genet (MGG).

[70] Hellens RP, Edwards EA, Leyland NR, Bean S, Mullineaux PM (2000). pGreen: a versatile and flexible binary Ti vector for Agrobacterium-mediated plant transformation. Plant Mol Biol.

[71] Clough SJ, Bent AF (1998). Floral dip: a simplified method for Agrobacterium-mediated transformation of *Arabidopsis thaliana*. Plant J.

[72] Murashige T, Skoog F (1962). A revised medium for rapid growth and bio assays with tobacco tissue cultures. Physiol Plant.

[73] Schmittgen TD, Livak KJ (2008). Analyzing real-time PCR data by the comparative C(T) method. Nat Protoc.

[74] Livak KJ, Schmittgen TD (2001). Analysis of relative gene expression data using real-time quantitative PCR and the 2(−Delta Delta C(T)) method. Methods.

[75] Arvidsson S, Kwasniewski M, Riano-Pachon DM, Mueller-Roeber B (2008). QuantPrime—a flexible tool for reliable high-throughput primer design for quantitative PCR. BMC Bioinform.

