# Supplementary material for: Simultaneous knockdown of six non-family genes using a single synthetic RNAi fragment in Arabidopsis thaliana
Source: Plant Methods. 2016 Feb 17;12:16. doi: 10.1186/s13007-016-0116-8 (PMC4756541; doi:10.1186/s13007-016-0116-8)

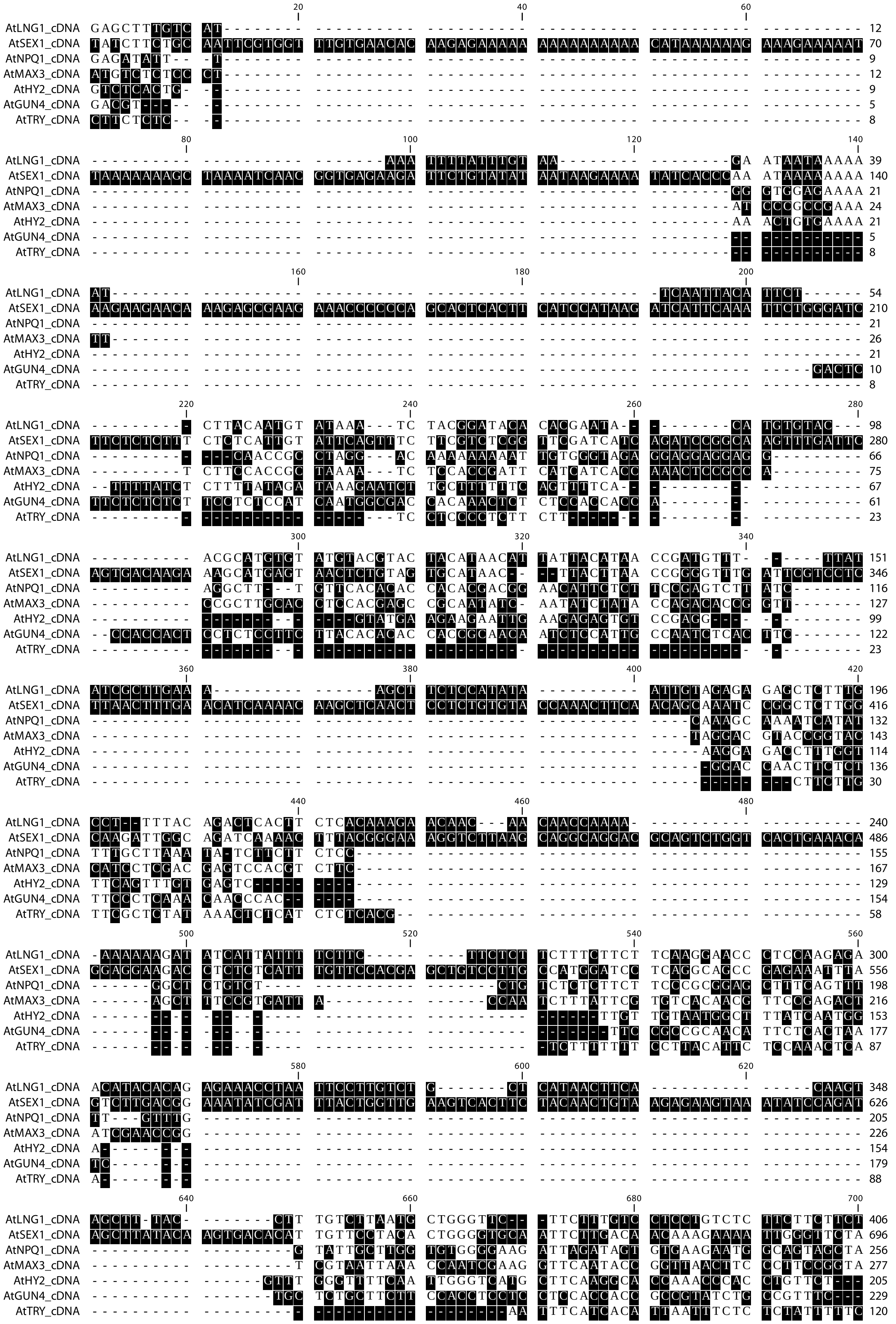

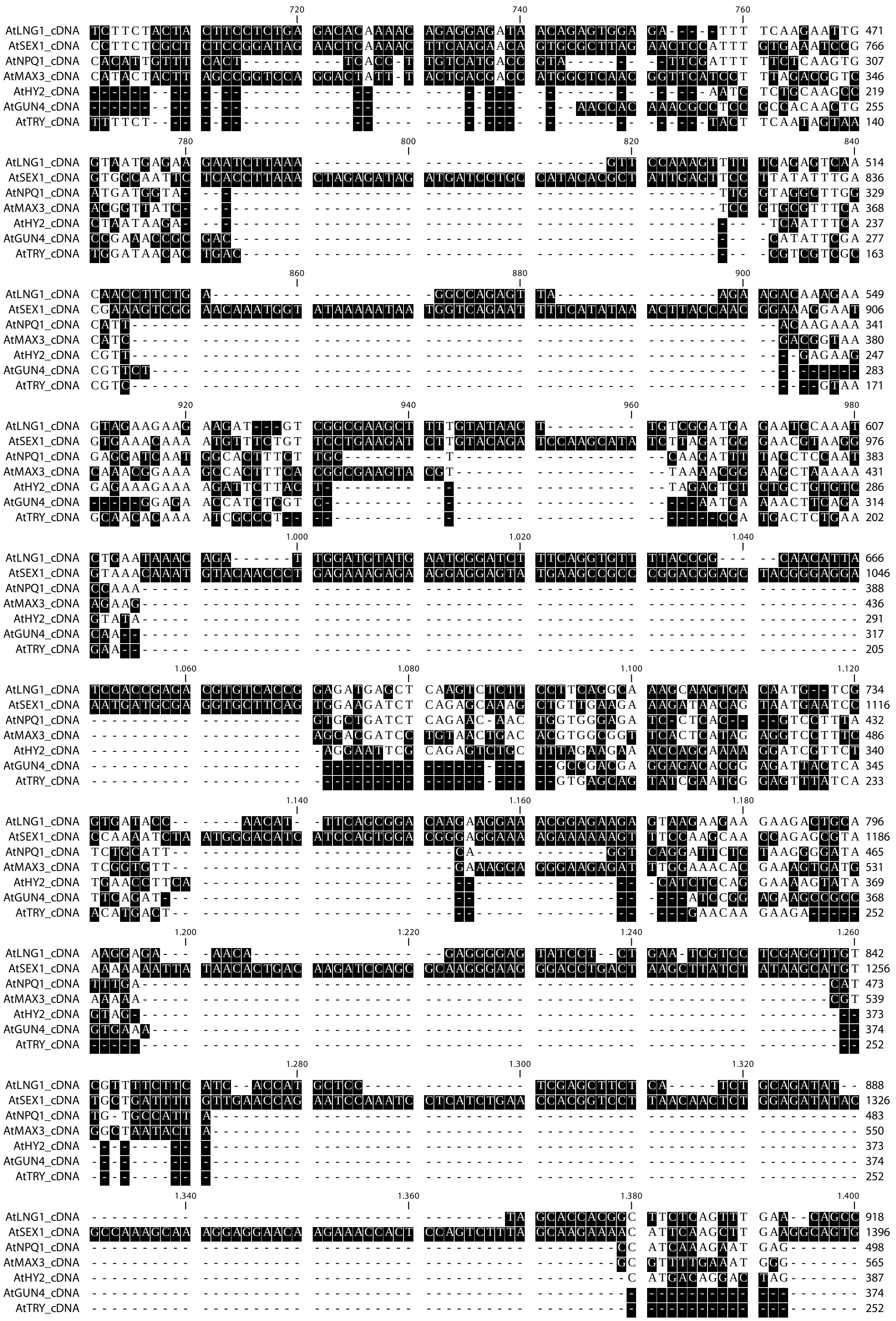
AtLNG1_cDNA
AtSEX1_cDNA
AtNPQ1_cDNA
AtMAX3_cDNA
AtHY2_cDNA
AtGUN4_cDNA
AtTRY_cDNA

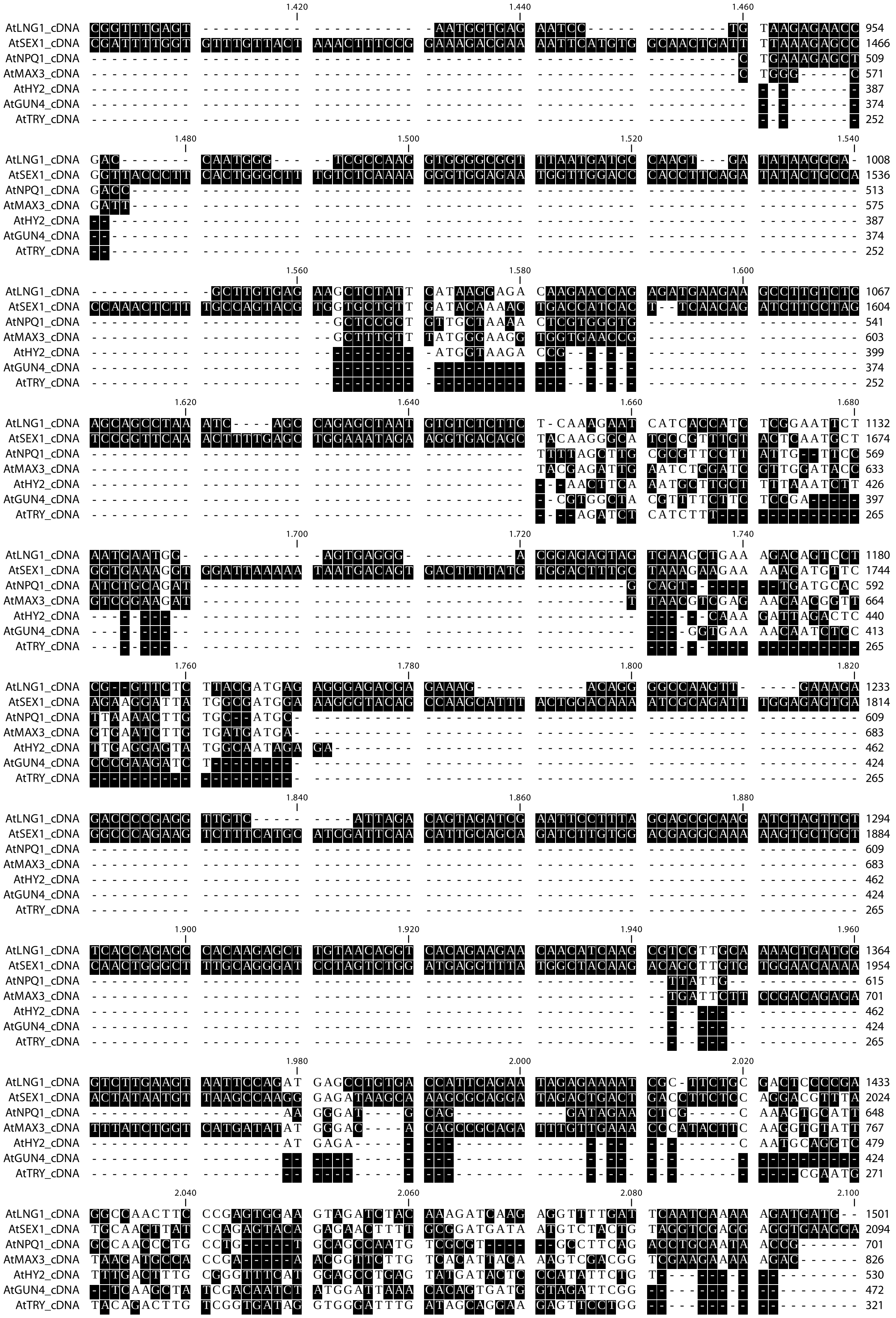
AtLNG1_cDNA
AtSEX1_cDNA
AtNPQ1_cDNA
AtMAX3_cDNA
AtHY2_cDNA
AtGUN4_cDNA
AtTRY_cDNA

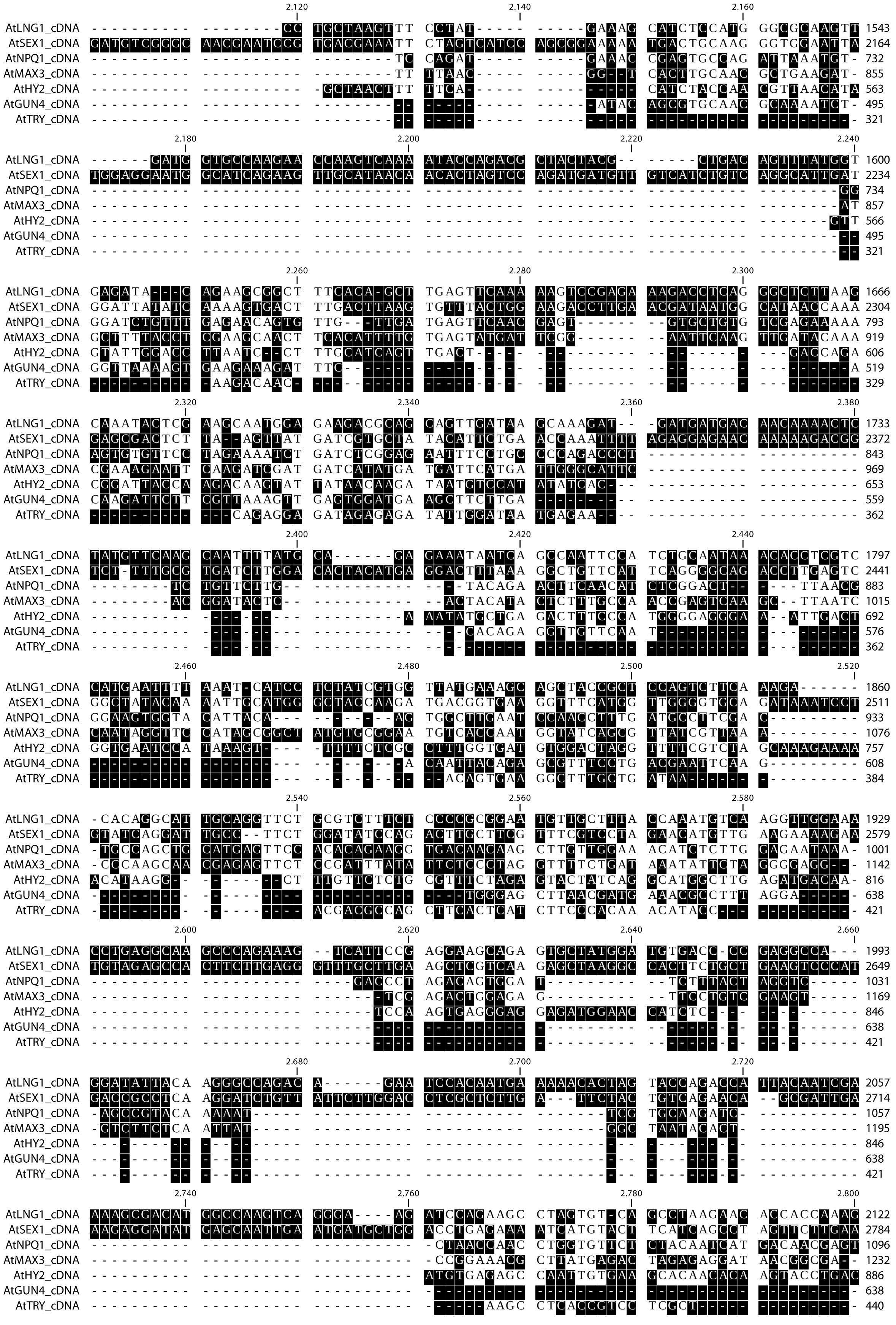

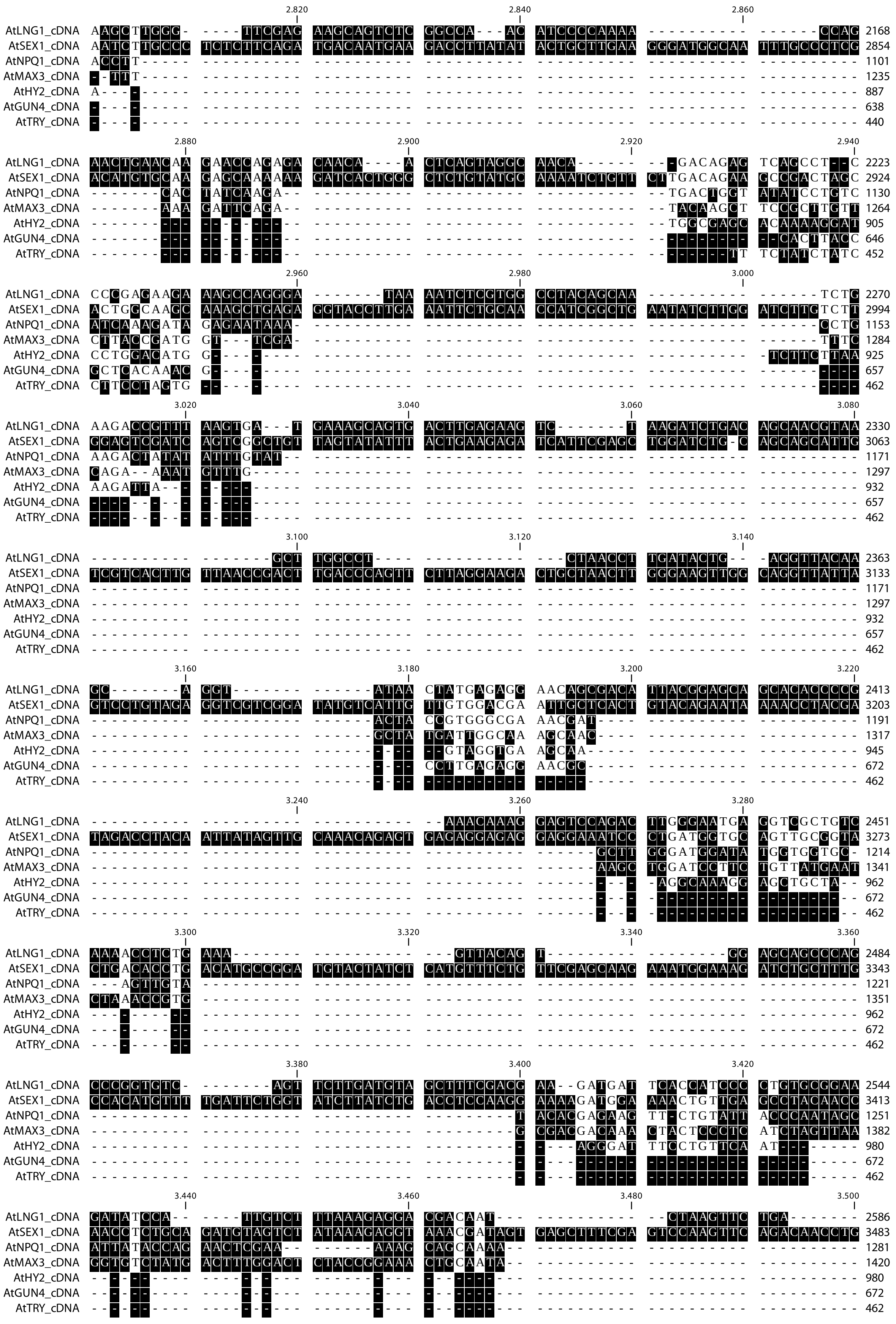

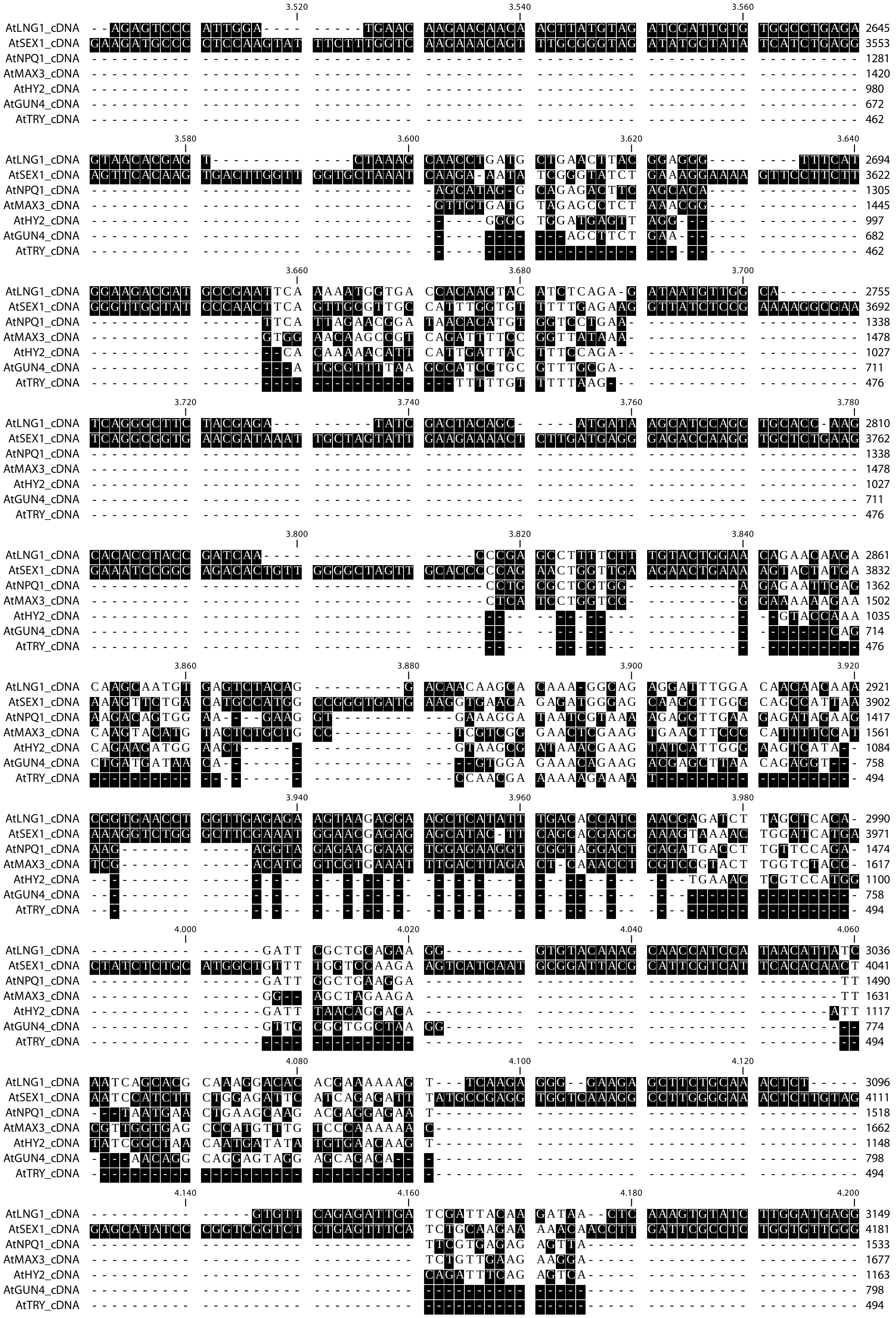
AtLNG1_cDNA
AtSEX1_cDNA
AtNPQ1_cDNA
AtMAX3_cDNA
AtHY2_cDNA
AtGUN4_cDNA
AtTRY_cDNA

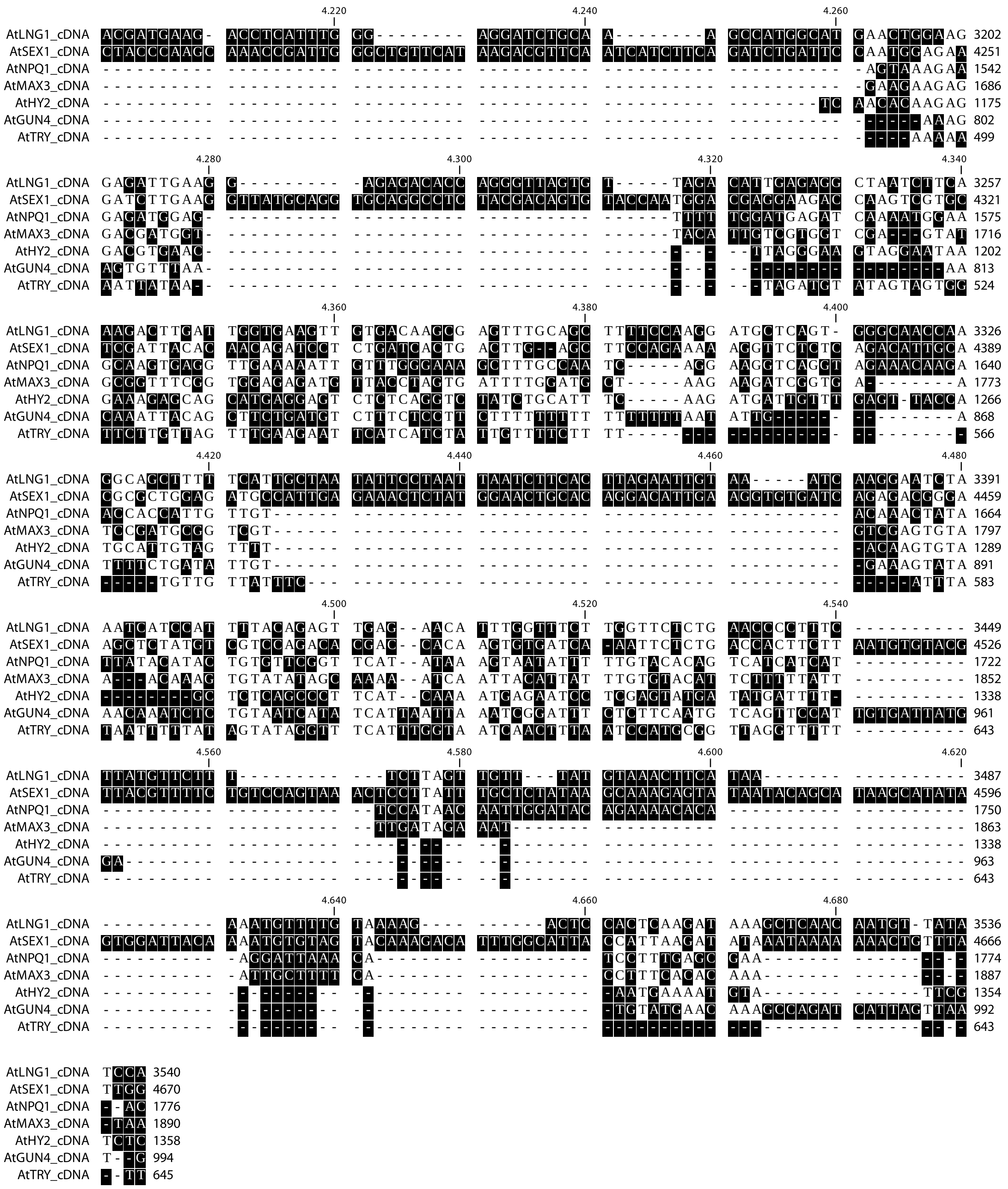

Supplement: Supplementary file 1 — 10.1186/s12014-016-9104-2 Alignment of cDNAs of AtHY2, AtTRY, AtLNG1, AtNPQ1, AtSEX1, AtMAX3 and AtGUN4. [file 13007_2016_116_MOESM1_ESM.pdf]
