# Supplementary material for: Simultaneous knockdown of six non-family genes using a single synthetic RNAi fragment in Arabidopsis thaliana
Source: Plant Methods. 2016 Feb 17;12:16. doi: 10.1186/s13007-016-0116-8 (PMC4756541; doi:10.1186/s13007-016-0116-8)
Supplement: Supplementary file 2 — 10.1186/s12014-016-9104-2 PCR primers used in this study. [file 13007_2016_116_MOESM2_ESM.docx]

**Supplemental Table 1.** Primers used in this study. Gene specific cloning primer sequences for GSTs were obtained from the CATMA database [66]. The underlined sequence (5’-CACC-3’) was introduced into the forward primer sequence to facilitate site directed cloning into pENTR^TM^/D-TOPO. Quantitative RT-PCR primers were designed using QUANTPRIME [73]. The pAGRIKOLA sequencing primers derived from the AGRIKOLA website [20]. The GeneArt® Primer and Construct Design Tool (http://www.thermofisher.com/order/oligoDesigner) was used to design primers for the assembly of the synthetic RNAi fragments.

| **Primer** | **5’-3’ Sequences** |
| --- | --- |
|  |  |
| **Cloning Primers – Single RNAi Fragments** | |
|  |  |
| AtHY2_RNAi_fw | CACCAGTCAGATTTCAGAGTCATCAACAC; |
| AtHY2_RNAi_rv | AATCTCTGTTGATTAGGTAGAGACG |
| AtLNG1_RNAi_fw | CACCAGTATCCTCTGAATCGTCCTCG; |
| AtLNG1_RNAi_rv | CACCTTGGCGACCCATTG |
| AtTRY_RNAi_fw | CACCTGGGATTTGATAGCAGGA; |
| AtTRY_RNAi_rv | CACTAGGAAGGATAGATAGAAAAGC |
| AtMAX3_RNAi_fw | CACCGATTGGCAAAGCAACAAG; |
| AtMAX3_RNAi_rv | CAATGTAACCATCGTCCTCT |
| AtNPQ1_RNAi_fw | CACCCCAGAACTCGAAAAAGCAG; |
| AtNPQ1_RNAi_rv | GTACAACAATGGTGGTTCTT |
| AtSEX1_RNAi_fw | CACCGCAATTTGCCCTCGACAT; |
| AtSEX1_RNAi_rv | GCCGACTGATCGACTCCAAG |
| AtGUN4_RNAi_fw | CACCCTTCAGACAAGCCGACGAGG; |
| AtGUN4_RNAi_rv | AAACGCAGGATGGCTTAAAA |
|  |  |
| **qRT-PCR** | |
|  |  |
| AtHY2 | ATCAGGCATGGCTTGAGATGAC; |
|  | TGGCTCTCACATGAGATGGTTCC |
| AtLNG1 | TTATCCACCGAGACGTGTCACC; |
|  | CATTGTCACTTGCTTTGCCTGAAG |
| AtTRY | GTACAGACTTGTCGGTGATAGGTG; |
|  | GAAGCTGGCGTCGTTTATCAGC |
| AtMAX3 | TCGTTGGTGAGCCCATGTTTGTC; |
|  | TCTCCACCGAAACCGCATACTC |
| AtNPQ1 | GCTTGCGCGTTCCTTATTGTTCC; |
|  | ATGCACTTTGCGAGTTCTATCCTG |
| AtSEX1 | CAACCTTGATTCGCCTCTGGTG; |
|  | ACGTCTTATGAACAGCCCAATCG |
| AtGUN4 | CTGCCGTTTCAACCACAAACGC; |
|  | ACGTCGAATATGGTCGCGGTTTC |
| AtACT2 | CCAGAAGGATGCATATGTTGGTGA; |
|  | GAGGAGCCTCGGTAAGAAGA |
|  |  |
| **pAGRIKOLA Sequencing Primers** | |
|  |  |
| AGRI51 | CAACCACGTCTTCAAAGCAA |
| AGRI56 | CTGGGGTACCGAATTCCTC |
| AGRI64 | CTTGCGCTGCAGTTATCATC |
| AGRI69 | AGGCGTCTCGCATATCTCAT |
| Cat_Intron | AATTGGGTTCGAAATCGATAAGC |
| Pdk_Intron | TCTTCTTCGTCTTACACATCACTTG |
|  |  |
| **Primers – Assembling Multiple RNAi Fragments** | |
|  |  |
| *(a) Construct HTLNSM* | |
|  |  |
| a_HY2_FW | GCGCAGCGGCGGCCGCGCTGATACCGCCGCAGTCAGATTT CAGAGTCATCAACA |
| a_HY2_RV | TGCTATCAAATCCCAAATCTCTGTTGATTAGGTAGAGAC |
| a_TRY_FW | TAATCAACAGAGATTTGGGATTTGATAGCAGGAAGAGTT |
| a_TRY_RV | GATTCAGAGGATACTCACTAGGAAGGATAGATAGAAAAG |
| a_LNG1_FW | CTATCCTTCCTAGTGAGTATCCTCTGAATCGTCCTCGAG |
| a_LNG1_RV | TTTTTCGAGTTCTGGCACCTTGGCGACCCATTGGTCGGT |
| a_NPQ1_FW | TGGGTCGCCAAGGTGCCAGAACTCGAAAAAGCAGCAAAA |
| a_NPQ1_RV | TCGAGGGCAAATTGCGTACAACAATGGTGGTTCTTGTTT |
| a_SEX1_FW | CCACCATTGTTGTACGCAATTTGCCCTCGACATGTGCAA |
| a_SEX1_RV | GTTGCTTTGCCAATCGCCGACTGATCGACTCCAAGACAA |
| a_MAX3_FW | AGTCGATCAGTCGGCGATTGGCAAAGCAACAAGCTGGAT |
| a_MAX3_RV | GCTCACTGACTTTAATTAACTGCGGCGAGGCAATGTAACC ATCGTCCTCTTCTT |
|  | |
| *(b) Construct NLSHMT* | |
|  |  |
| b_NPQ1_FW | GCGCAGCGGCGGCCGCGCTGATACCGCCGCCCAGAACTCG AAAAAGCAGCAAAA |
| b_NPQ1_RV | GATTCAGAGGATACTGTACAACAATGGTGGTTCTTGTTT |
| b_LNG1_FW | CCACCATTGTTGTACAGTATCCTCTGAATCGTCCTCGAG |
| b_LNG1_RV | TCGAGGGCAAATTGCCACCTTGGCGACCCATTGGTCGGT |
| b_SEX1_FW | TGGGTCGCCAAGGTGGCAATTTGCCCTCGACATGTGCAA |
| b_SEX1_RV | CTCTGAAATCTGACTGCCGACTGATCGACTCCAAGACAA |
| b_HY2_FW | AGTCGATCAGTCGGCAGTCAGATTTCAGAGTCATCAACA |
| b_HY2_RV | GTTGCTTTGCCAATCAATCTCTGTTGATTAGGTAGAGAC |
| b_MAX3_FW | TAATCAACAGAGATTGATTGGCAAAGCAACAAGCTGGAT |
| b_MAX3_RV | TGCTATCAAATCCCACAATGTAACCATCGTCCTCTTCTT |
| b_TRY_FW | ACGATGGTTACATTGTGGGATTTGATAGCAGGAAGAGTT |
| b_TRY_RV | GCTCACTGACTTTAATTAACTGCGGCGAGGCACTAGGAAG GATAGATAGAAAAG |
|  |  |
| *(c) Construct LNTMHS* | |
|  |  |
| c_LNG1_FW | GCGCAGCGGCGGCCGCGCTGATACCGCCGCAGTATCCTC TGAATCGTCCTCGAG |
| c_LNG1_RV | same as a_LNG1_RV |
| c_NPQ1_FW | same as a_NPQ1_FW |
| c_NPQ1_RV | TGCTATCAAATCCCAGTACAACAATGGTGGTTCTTGTTT |
| c_TRY_FW | CCACCATTGTTGTACTGGGATTTGATAGCAGGAAGAGTT |
| c_TRY_RV | GTTGCTTTGCCAATCCACTAGGAAGGATAGATAGAAAAG |
| c_MAX3_FW | CTATCCTTCCTAGTGGATTGGCAAAGCAACAAGCTGGAT |
| c_MAX3_RV | CTCTGAAATCTGACTCAATGTAACCATCGTCCTCTTCTT |
| c_HY2_FW | ACGATGGTTACATTGAGTCAGATTTCAGAGTCATCAACA |
| c_HY2_RV | TCGAGGGCAAATTGCAATCTCTGTTGATTAGGTAGAGAC |
| c_SEX1_FW | TAATCAACAGAGATTGCAATTTGCCCTCGACATGTGCAA |
| c_SEX1_RV | GCTCACTGACTTTAATTAACTGCGGCGAGGGCCGACTGAT CGACTCCAAGACAA |
|  |  |
| *(d) Construct GHTLNSM* | |
|  |  |
| d_GUN4_FW | GCGCAGCGGCGGCCGCGCTGATACCGCCGCCTTCAGACAA GCCGACGAGGAGAC |
| d_GUN4_RV | CTCTGAAATCTGACTAAACGCAGGATGGCTTAAAACGCA |
| d_HY2_FW | AGCCATCCTGCGTTTAGTCAGATTTCAGAGTCATCAACA |
| d_HY2_RV | same as a_HY2_RV |
| d_TRY_FW | same as a_TRY_FW |
| d_TRY_RV | same as a_TRY_RV |
| d_LNG1_FW | same as a_LNG1_FW |
| d_LNG1_RV | same as a_LNG1_RV |
| d_NPQ1_FW | same as a_NPQ1_FW |
| d_NPQ1_RV | same as a_NPQ1_RV |
| d_SEX1_FW | same as a_SEX1_FW |
| d_SEX1_RV | same as a_SEX1_RV |
| d_MAX3_FW | same as a_MAX3_FW |
| d_MAX3_RV | same as a_MAX3_RV |
|  |  |
| *(e) Construct NLSHMTG* | |
|  |  |
| e_NPQ1_FW | same as b_NPQ1_FW |
| e_NPQ1_RV | same as b_NPQ1_RV |
| e_LNG1_FW | same as b_LNG1_FW |
| e_LNG1_RV | same as b_LNG1_RV |
| e_SEX1_FW | same as b_SEX1_FW |
| e_SEX1_RV | same as b_SEX1_RV |
| e_HY2_FW | same as b_HY2_FW |
| e_HY2_RV | same as b_HY2_RV |
| e_MAX3_FW | same as b_MAX3_FW |
| e_MAX3_RV | same as b_MAX3_RV |
| e_TRY_FW | same as b_TRY_FW |
| e_TRY_RV | TCGGCTTGTCTGAAGCACTAGGAAGGATAGATAGAAAAG |
| e_GUN4_FW | CTATCCTTCCTAGTGCTTCAGACAAGCCGACGAGGAGAC |
| e_GUN4_RV | GCTCACTGACTTTAATTAACTGCGGCGAGGAAACGCAGGA TGGCTTAAAACGCA |
|  |  |
| *(f) Construct LNGTMHS* | |
|  |  |
| f_LNG1_FW | same as c_LNG1_FW |
| f_LNG1_RV | same as c_LNG1_RV |
| f_NPQ1_FW | same as c_NPQ1_FW |
| f_NPQ1_RV | TCGGCTTGTCTGAAGGTACAACAATGGTGGTTCTTGTTT |
| f_GUN4_FW | CCACCATTGTTGTACCTTCAGACAAGCCGACGAGGAGAC |
| f_GUN4_RV | TGCTATCAAATCCCAAAACGCAGGATGGCTTAAAACGCA |
| f_TRY_FW | AGCCATCCTGCGTTTTGGGATTTGATAGCAGGAAGAGTT |
| f_TRY_RV | same as c_TRY_RV |
| f_MAX3_FW | same as c_MAX3_FW |
| f_MAX3_RV | same as c_MAX3_RV |
| f_HY2_FW | same as c_HY2_FW |
| f_HY2_RV | same as c_HY2_RV |
| f_SEX1_FW | same as c_SEX1_FW |
| f_SEX1_RV | same as c_SEX1_RV |
|  |  |
| **Assembly Proof PCR / Cloning Primers – Multiple RNAi Fragments** | |
|  |  |
| *(a) Construct HTLNSM* | |
|  |  |
| AtHY2_for | same as AtHY2_RNAi_fw |
| AtMAX3_rev | same as AtMAX3_RNAi_rv |
|  |  |
| *(b) Construct NLSHMT* | |
|  |  |
| AtNPQ1_for | same as AtNPQ1_RNAi_fw |
| AtTRY_rev | same as AtTRY_RNAi_rv |
|  |  |
| *(c) Construct LNTMHS* | |
|  |  |
| AtLNG1_for | same as AtLNG1_RNAi_fw |
| AtSEX1_rev | same as AtSEX1_RNAi_rv |
|  |  |
| *(d) Construct GHTLNSM* | |
|  |  |
| AtGUN4_for | same as AtGUN4_RNAi_fw |
| AtMAX3_rev | same as AtMAX3_RNAi_rv |
|  |  |
| *(e) Construct NLSHMTG* | |
|  |  |
| AtNPQ1_for | same as AtNPQ1_RNAi_fw |
| AtGUN4_rev | same as AtGUN4_RNAi_rv |
|  |  |
| *(f) Construct LNGTMHS* | |
|  |  |
| AtLNG1_for | same as AtLNG1_RNAi_fw |
| AtSEX1_rev | same as AtSEX1_RNAi_rv |
